# Supplementary material for: Cell-type specific concentration regulation of the basal transcription factor TFIIH in XPBy/y mice model
Source: Cancer Cell Int. 2019 Sep 10;19:237. doi: 10.1186/s12935-019-0945-4 (PMC6734240; doi:10.1186/s12935-019-0945-4)
Supplement: Supplementary file 1 — Additional file 1: Figure S1. Western blot showing the expression of TFIIH subunits in whole cell extract of WT and XPB-YFP stably-expressing MDF cells. α-tub serves as a loading control. Figure S2. A Simplified scheme representing the image post-processing and analysis pipeline. First, living organotypic tissue slices or cultured cells (Xpby/y mouse) are imaged by confocal microscopy, typically over a 230 × 230 × 30 µm region. Second, the resulting image stacks are processed via deconvolution to restore the spatial distribution of the fluorescence signal. Third, using the same software, fluorescence intensity iso-surfaces are generated to calculate the volume occupied by the XPB-YFP signal of well segmented, non-truncated cell nuclei. Finally, the total XPB-YFP derived fluorescence per cell was estimated by integrating over each of these volumes. B. Side by side comparison of the XPB-YFP signal in living chondrocytes (Xpby/y mouse) and the fluorescence of diluted YFP recombinant protein obtained with identical imaging parameters. Scale bar represents 20 µm. C. Single cell fluorescence quantification of DAPI stained cycling murine chondrocytes via deconvolved 3D imaging as described in the first panel. The graph is a histogram of the resulting total DAPI signal per cell (in arbitrary units). After normalizing the lowest peak to 1, the second peak is found at 2 ± 0.05. D. Scatter plot of the normalized total DAPI signal per cell versus the reconstructed nuclear volume. The dotted line represents a constant DAPI concentration. [file 12935_2019_945_MOESM1_ESM.docx]

**ADDITIONAL FILE**

**
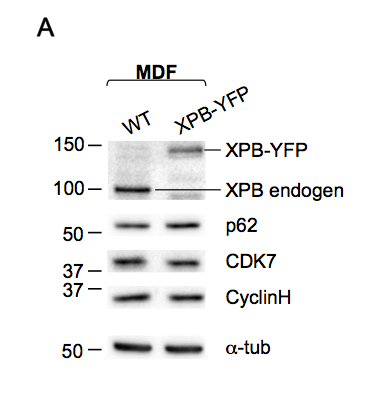
**

**Figure S1.** Western blot showing the expression of TFIIH subunits in whole cell extract of WT and XPB-YFP stably-expressing MDF cells. α-tub serves as a loading control.

**
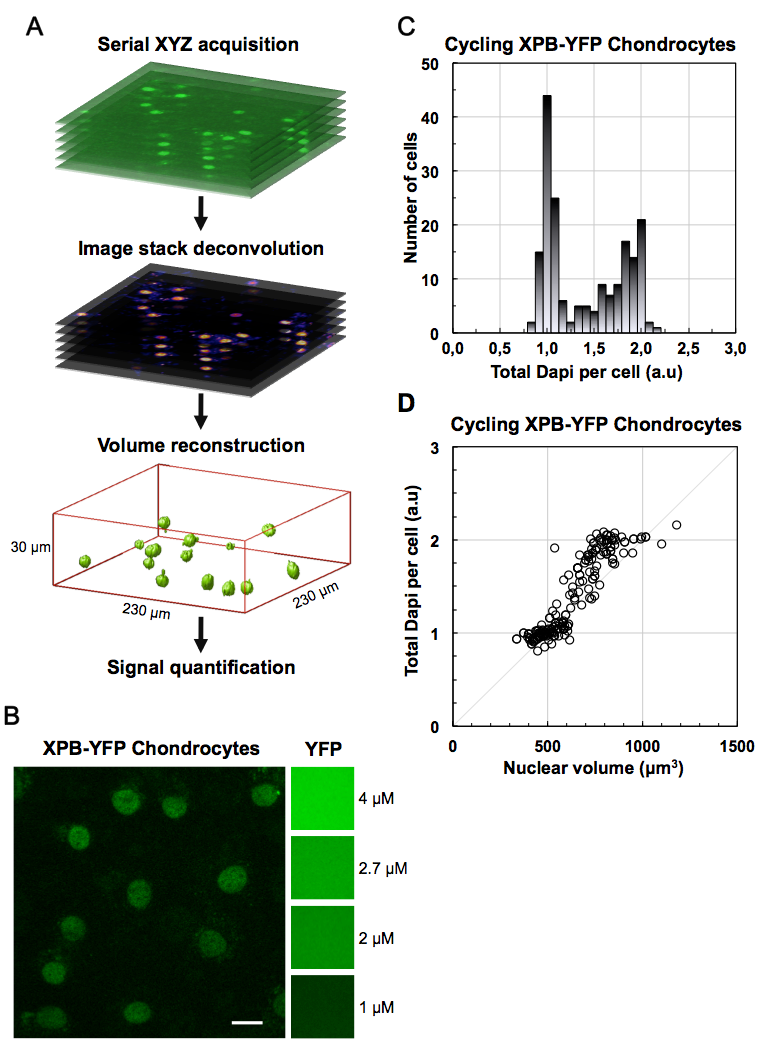
**

**Figure S2. A** Simplified scheme representing the image post-processing and analysis pipeline. First, living organotypic tissue slices or cultured cells (Xpb^y/y^ mouse) are imaged by confocal microscopy, typically over a 230x230x30 µm region. Second, the resulting image stacks are processed via deconvolution to restore the spatial distribution of the fluorescence signal. Third, using the same software, fluorescence intensity iso-surfaces are generated to calculate the volume occupied by the XPB-YFP signal of well segmented, non-truncated cell nuclei. Finally, the total XPB-YFP derived fluorescence per cell was estimated by integrating over each of these volumes. **B**. Side by side comparison of the XPB-YFP signal in living chondrocytes (Xpb^y/y^ mouse) and the fluorescence of diluted YFP recombinant protein obtained with identical imaging parameters. Scale bar represents 20 µm. **C.** Single cell fluorescence quantification of DAPI stained cycling murine chondrocytes via deconvolved 3D imaging as described in the first panel. The graph is a histogram of the resulting total DAPI signal per cell (in arbitrary units). After normalizing the lowest peak to 1, the second peak is found at 2±0.05. **D.** Scatter plot of the normalized total DAPI signal per cell versus the reconstructed nuclear volume. The dotted line represents a constant DAPI concentration.
